# Tissue-specific temperature dependence of RNA editing levels in zebrafish

**DOI:** 10.1186/s12915-023-01738-4

**Published:** 2023-11-20

**Authors:** Wenhao Li, Mengdi Bu, Ruiqin Hu, Shouwen Jiang, Liangbiao Chen, George N. Somero

**Affiliations:** 1 Key Laboratory of Exploration and Utilization of Aquatic Genetic Resources, Ministry of Education, College of Fisheries and Life Sciences, Shanghai Ocean University, Shanghai, 201306 China; 2 International Research Center for Marine Biosciences, Ministry of Science and Technology, College of Fisheries and Life Sciences, Shanghai Ocean University, Shanghai, 201306 China; 3 Department of Biology, Hopkins Marine Station, Stanford University, Pacific Grove, CA 93950 USA

**Keywords:** Acclimation, Adenosine deaminase acting on RNA (ADAR), Corresponding states, RNA, RNA editing, Temperature, Zebrafish

## Abstract

**Background:**

RNA editing by *a*denosine *d*eaminase *a*cting on *R*NA (ADAR) occurs in all metazoans and fulfils several functions. Here, we examined effects of acclimation temperature (27 °C, 18 °C,13 °C) on editing patterns in six tissues of zebrafish (*Danio rerio*).

**Results:**

Sites and total amounts of editing differed among tissues. Brain showed the highest levels, followed by gill and skin. In these highly edited tissues, decreases in temperatures led to large increases in total amounts of editing and changes in specific edited sites. Gene ontology analysis showed both similarities (e.g., endoplasmic reticulum stress response) and differences in editing among tissues. The majority of edited sites were in transcripts of transposable elements and the 3′UTR regions of protein coding genes. By experimental validation, translation efficiency was directly related to extent of editing of the 3′UTR region of an mRNA.

**Conclusions:**

RNA editing increases 3′UTR polymorphism and affects efficiency of translation. Such editing may lead to temperature-adaptive changes in the proteome through altering relative amounts of synthesis of different proteins.

**Supplementary Information:**

The online version contains supplementary material available at 10.1186/s12915-023-01738-4.

## Background

RNA editing through deamination reactions, a process by which a specific RNA base encoded in the DNA is converted to a different base (adenosine to inosine or cytosine to uridine), is a phylogenetically ancient process thought to have arisen in the common ancestor of contemporary metazoans [1–4]. The most common type of RNA editing, conversion of A-to-I, is catalyzed by the enzyme *a*denosine *d*eaminase *a*cting on *R*NA (ADAR), which edits both co-transcriptionally and post-transcriptionally [5–7]. Because I is read as a G during formation of base pairs, e.g., during translation, this type of editing is commonly referred to as A-to-G editing. RNA editing through deamination of adenosine (hereafter, RNA editing) is one of a family of changes in RNA that have been termed “epitranscriptomic” alterations [8] in recognition of the fact that diverse RNAs can be generated co- or post-transcriptionally without any alteration in DNA sequence. The full suite of functions of RNA editing remains to be fully elucidated, but available data show that RNA editing plays diverse and important roles. For example, editing in coding regions (CDS), termed recoding editing, may lead to adaptive change in the sequences of proteins involved in neural function in mammals [9] and cephalopods (octopus) [1, 10]. However, in view of the fact that the large majority of editing occurs outside of coding regions, especially in 3′ untranslated regions (3′UTRs), introns, and intergenic regions, the biological significance of the editing process is likely to include advantageous effects beyond those that involve recoding. Known or hypothesized effects include those on RNA turnover, RNA interference (editing of microRNAs), RNA splicing (editing-induced changes in splice junctions), RNA stability [11], and suppression of repetitive elements, e.g., Alu elements [3, 12]. RNA editing also has recently been discovered to be a widespread mechanism of RNA post-transcriptional regulation across many tissues or developmental stages in mammals and other animals, including zebrafish [13].

Whatever the diverse functional consequences of RNA editing are, changes in cell temperature would be expected to significantly alter the amounts and sites of editing due to direct temperature effects on RNA higher-order structures and on the activity and turnover of ADAR [4, 10, 14–17]. Pervasive effects of temperature on editing would be expected because of the thermal sensitivity of secondary and tertiary structures of RNA [14, 18–22], both of which are important in regulating the editing process. For editing to occur by ADAR, the edited adenosine must occur within a region of double-stranded RNA (dsRNA) that is at least 23 base pairs in length, albeit the double-strandedness of this essential set of base pairs must be imperfect [3, 18]. Tertiary structural elements, e.g., pseudoknots, that govern RNA editing are also likely to be highly sensitive to temperature [14]. To a first approximation then, the stabilization of RNA secondary and tertiary structures that would be expected to occur at reduced temperatures would be expected to foster increased RNA editing. Temperature also affects the activity and cellular levels of ADAR [14], so even in the absence of a change in higher orders of RNA structure, some temperature dependence of editing would be expected.

Despite the fact that RNA editing is likely to be highly sensitive to changes in temperature, the occurrence and functional roles of editing in response to changes in temperature and other abiotic environmental factors remain largely unexplored. Here, to elucidate the patterns and potential functional importance of RNA editing in teleost fish under cold stress, we report the results of a study of temperature acclimation (27 °C, 18 °C, and 13 °C) on RNA editing patterns in the eurythermal zebrafish (*Danio rerio*). This genomically well-characterized species provides a powerful study system for examining the interactions of temperature and RNA editing across a wide range of temperatures and cell types. Our study examined six tissues (brain, gill, muscle, skin, ovary, and liver) that play diverse roles in the fish’s physiology. We characterize the sites of RNA editing in different genomic regions and show that editing is relatively rare in CDS, but abundant in intergenic and 3′UTR regions at all temperatures. Tissue-specific editing patterns are present at all temperatures and changes in temperature are shown to have a strong effect on amounts and types of editing. Importantly, the efficiency with which a mRNA is translated is directly related to the extent of ADAR-driven editing in the 3′UTR. Editing is proposed to play an important role in the metabolic re-organization that commonly occurs in ectotherms like fishes during temperature acclimation.

## Results

### Characterization of the editome of zebrafish

**Fig. 1:**
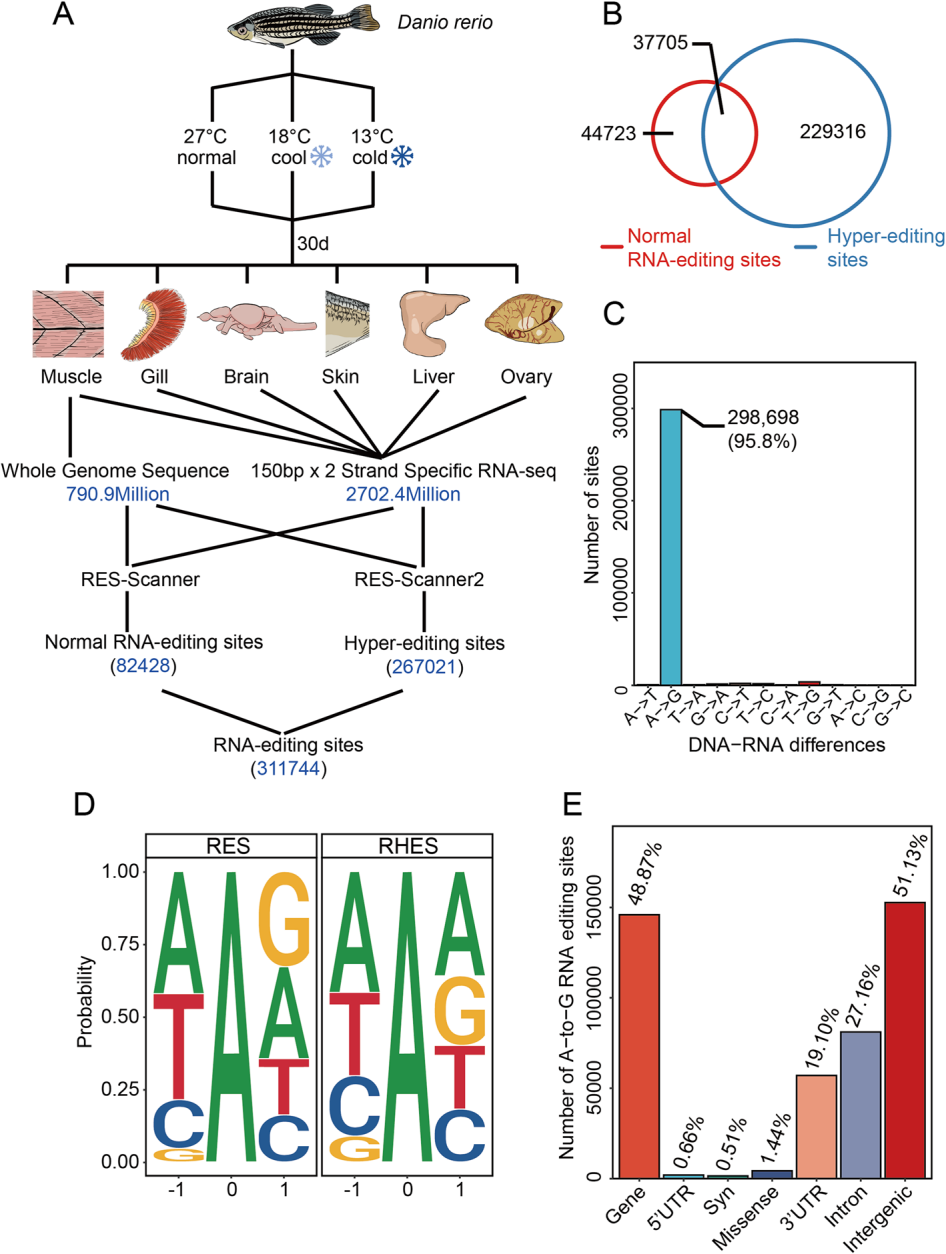
Experimental strategies and the RNA editome in zebrafish. **A** Experimental design for RNA editing study in cold-acclimated zebrafish. **B** Venn diagram showing the distribution of candidate normal and hyper-edited sites. **C** Relative abundance of each editing type. A-to-G refers to A-to-I editing because I is read as a G during translation. **D** The preferred editing site deduced from the zebrafish editome. “RES” indicates normal *R*NA *e*dit *s*ites; “RHES” indicates *R*NA *h*yper-*e*dited *s*ites. **E** The relative abundance of editing in various genomic regions. Syn indicates synonymous CDS sites. Only females were used in analysis. Data from all acclimation groups were pooled to assess the relative prevalence of editing types

We performed both whole genome re-sequencing and strand-specific transcriptome sequencing for zebrafish acclimated to a typical holding temperature for the species (27 °C) and to temperatures that are either cool (18 °C) or cold (13 °C) relative to the species’ thermal tolerance range [23, 24]. Analyses of edited sites (the editome), which entailed comparing sequences of DNA with corresponding sequences of mRNA, were performed with six tissues of nine individuals (Fig. 1A). The pooled genomic DNA reads (790.9 million reads) and all RNA reads (2702.4 million) were mapped to the zebrafish reference genome (GRCz10). In total, 82,428 candidate normal RNA editing sites were detected by RES-scanner software [25], and 267,021 candidate hyper-editing sites were detected by RES-scanner2 software [26]. Hyper-editing sites occur in clusters and commonly have been greatly underestimated with earlier protocols for detecting RNA editing because these heavily edited complex sites are not easily aligned to the genome [2]. The two detection methods revealed 37,705 common editing sites, defined as sites where overlap of editing sites between normal RNA editing sites (RES-scanner) and hyper-editing sites (RES-scanner2) methods was detected (Fig. 1B). The number of hyper-editing sites identified was over 13 times larger than found in a previous study of this species [13]. Our findings thus support the hypothesis that hyper-editing sites account for the majority of editing sites [2] in zebrafish, rather than a fraction smaller than the normal RNA editing events as found in earlier work with this species [13]. Of the 12 types of editing that are possible, ADAR-catalyzed A-to-I editing is by far the most common class of editing across all tissues; 95.8% of the editings were canonical A-to-I base changes and other types of editing were rarely identified (Fig. 1C). We also observed a strong A-to-I peak in both the hyper-editing mode and normal editing mode (Additional file 1: Figure S1). In total, we identified 298,698 A-to-I editing sites in the six tissues (Additional file 2), which is a far larger number of sites than were identified in previous studies (64,167) [13]. The other classes of editing are rare and might be artifacts that were caused by sequencing or read-mapping errors [25]. Thus, we focused strictly on A-to-I (A-to-G) editing for further analysis.

We analyzed our data to identify the ADAR recognition motifs for the two types of ADAR-catalyzed editing we examined: normal (RES indicates normal *R*NA *e*dited *s*ites) and hyper-edited sites (RHES indicates *R*NA *h*yper-*e*dited sites). This entailed comparing the sequence context surrounding RES and RHES. The upstream nucleotide strongly favored adenosine in both RES and RHES. For the downstream nucleotide, the RES exhibited a preference for guanosine, whereas RHES favored adenosine (Fig. 1D).

The amounts of A-to-I editing found in all six tissues varied significantly among different genomic regions: 3′UTR (19.10%), 5′UTR (0.66%), CDS (1.95%), intergenic regions (51.13%), and introns (27.16%) (Fig. 1E). Thus, coding regions exhibited very small amounts of editing relative to non-coding regions, especially the 3′UTR, intergenic, and intron regions. Within the CDS, the majority of editing led to recoding: ~ 74% of the editing involved non-synonymous codons, whereas only ~ 26% of the editing involved synonymous codons. 51.13% of A-to-I editing sites occurred in an intergenic region, which is defined as the region between two genes and overlap primarily with transposable elements (TEs) and long intergenic non-coding RNAs (lincRNAs). 30.39% of A-to-I RNA editing sites were located in TEs in zebrafish (Additional file 1: Figure S2), in contrast with the overwhelming percentage (98%) of TE editing events, especially in Alu sequences (96%), in primates [27].

The percentage of A-to-I RNA editing sites, normalized to the frequency of each feature in the genome as a whole, also varied among genomic features. As shown in Additional file 1: Figure S3, the percentage of RNA editing sites in 3′UTR is apparently higher than in other genomics features.

### Tissue-specific editing patterns and categories of RNA editing across tissues

**Fig. 2:**
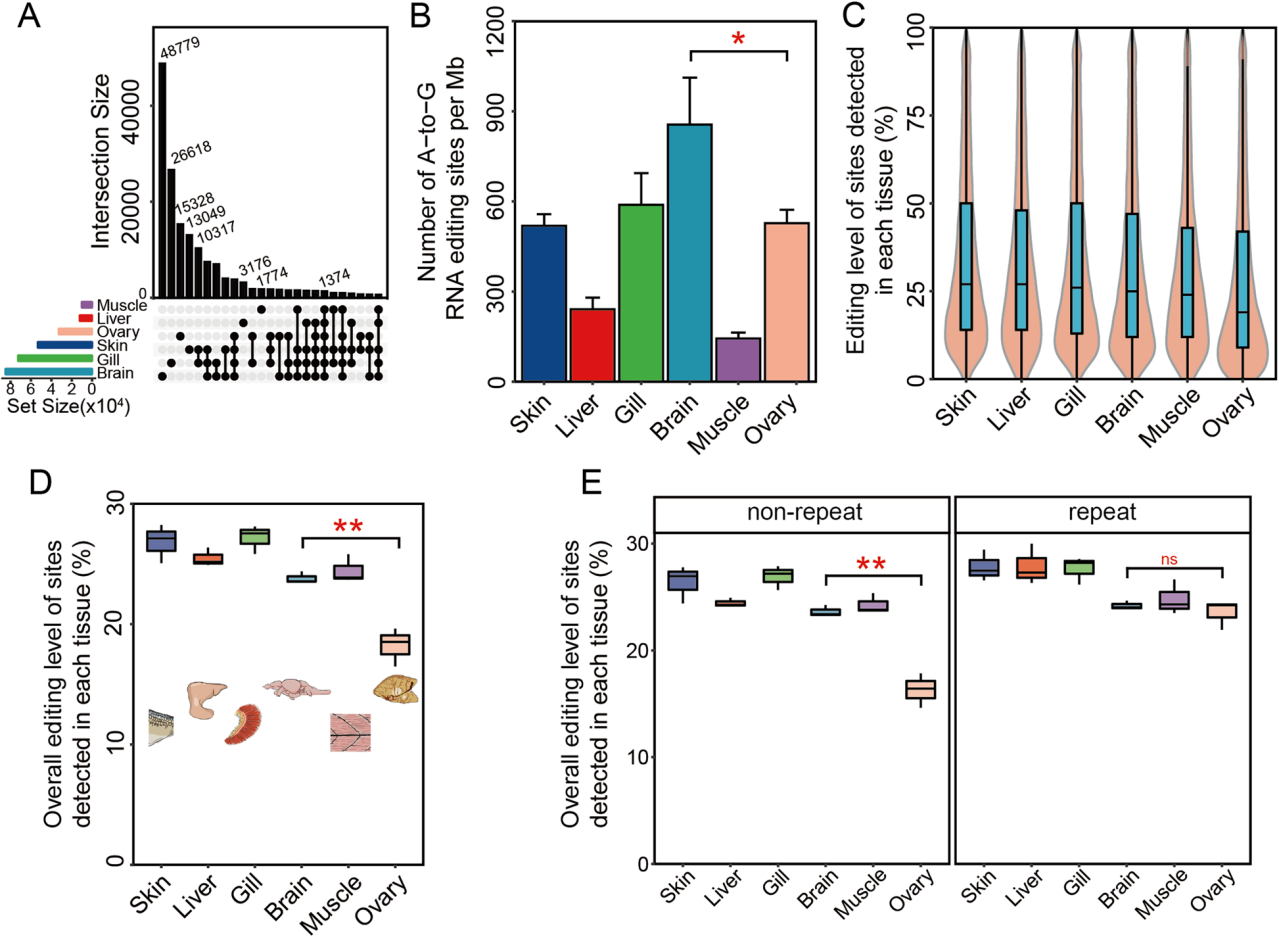
Characteristics of tissue-specific editomes in zebrafish. **A** Number of (commonly) edited sites of a single tissue or sites common to two or more tissues. This graphical UpSetR plot visualizes intersections of RNA editing sets in which the rows of the matrix represent the sets of tissues and columns represent their intersections (shared RNA editing behavior). Black filled circles indicate intersections; light gray circles indicate no intersection. The sizes of the intersections are shown by the bar graph above the matrix. **B** The per Mb density of edited sites for each tissue. Data are shown as the mean ± SD, *n* = 3. **C** Editing level at the detected edited sites in each tissue. Editing level was estimated as the number of G/the number of (G + A) in each editing site, in which G is the edited base count and A is the reference allele count. The shaded areas represent the distribution of editing levels. The horizonal lines in the boxes represent the median value of editing levels. **D, E** Overall editing levels of sites detected in each tissue of whole genome (**D**), and non-repeat DNA regions or repeat DNA regions (**E**) sites in various zebrafish tissues. Overall editing level is defined as the percentage of edited nucleotides at all known editing sites and was estimated as the total number of G in all editing sites/the total number of (A + G) in all editing sites. Statistical significance between brain and ovary for “Number of A-to-G RNA editing sites per Mb” and “Overall editing level of sites detected in each tissue (%)” were calculated using an unpaired *t*-test. *n* = 3, ns, not significant, *P < 0.05, **P < 0.01

To elucidate the tissue-specific editing patterns, we estimated the number of A-to-I RNA edited sites and editing levels across tissues using multiple methods. As shown by the UpSetR plot [28] in Fig. 2A, the great majority of edited sites are tissue-specific. Relatively few edited sites were shared among more than two tissues. The sharing of edited sites was highest in gill and skin (10,317 sites). The number of unique edited sites was highest in brain (48,779 sites), second highest in gill (26,618 sites), third highest in ovary (15,328), and lowest in muscle (1774 sites). The number of RNA edited sites has a remarkably linear correlation with the total number of mapped reads [29]. Therefore, an analysis that used the amount of mapped bases to normalize the number of A-to-I RNA edited sites was done (Fig. 2B). The data reveal substantial differences among tissues in the number of A-to-I edited sites per Mb (the total number of sites that were edited/million reads). Brain exhibited the greatest amounts of editing; gill, ovary, and skin had substantially lower amounts; liver and muscle exhibited the lowest amount of editing. Editing level of sites detected in each tissue (%) and overall editing level of sites detected in each tissue (%) were significantly lower in ovary than brain (Fig. 2C, D). The teleost genome contains various repeat DNA regions, such as transposon elements (TEs), and simple repeats. Zebrafish shows an overall 52.5% of repeat content [30]. Interestingly, the significantly lower levels of overall editing (%) of sites detected in ovary were mainly associated with the non-repeat DNA regions, rather the repeat DNAs (Fig. 2E). However, the high levels of editing observed in Fig. 2C–E could be attributed to our focus on the sites that the software packages [25, 26] detect in each tissue. And, in Fig. 2C–E, all the zero-edited sites in the specific tissues are excluded. We reanalyzed the editing level of each tissue based on the total editing sites combined from all tissues (a total of 298,698 sites) (Additional file 1: Figure S4). The editing levels observed in this analysis were significantly lower than those presented in Fig. 2C–E where the edited sites detected in the individual tissue were used.

The edited sites detected in Fig. 2C–E were reanalyzed using the “editing index,” which calculates the ratio of the number of A-to-G mismatches to the total coverage of adenosines in transposon element regions. We observed the same trend found by Buchumenski et al. [13], who employed this same quantification method (Additional file 1: Figure S5). In these analyses, the highest levels of editing index were detected in the zebrafish ovary.

### Principal components analysis of RNA editing patterns: evidence for temperature-driven editing

**Fig. 3:**
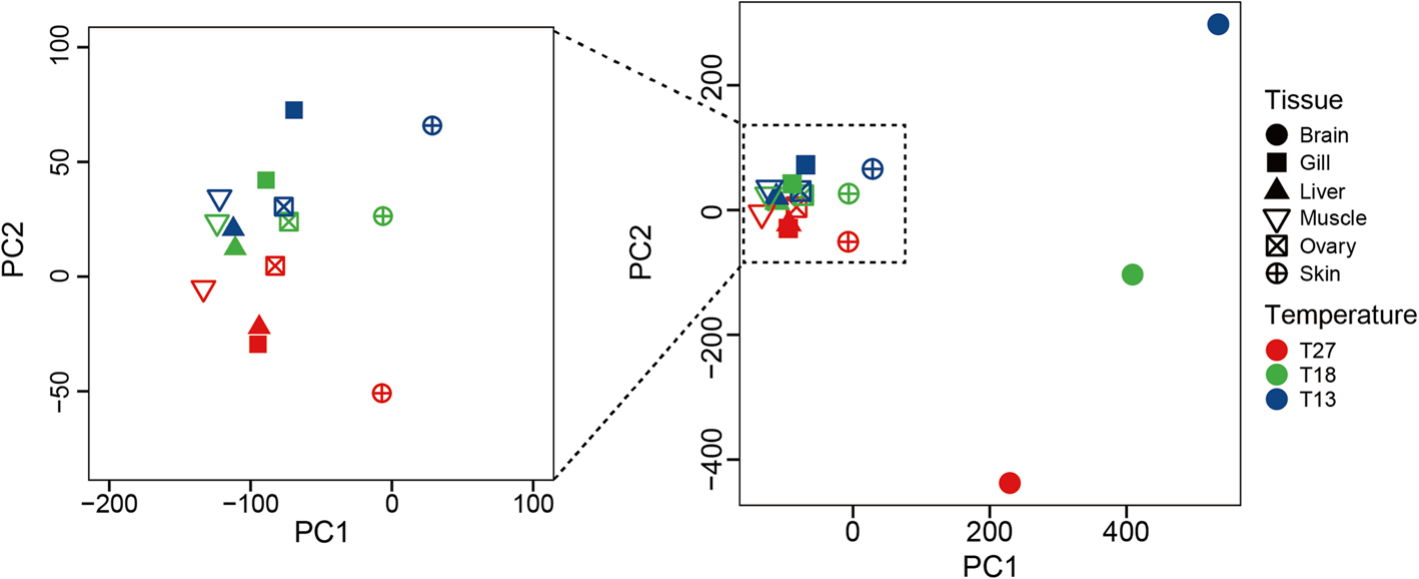
Principal component analysis of RNA editing across tissues from zebrafish acclimated to different temperatures

To distinguish the influences of acclimation temperature from those due to tissue type on patterns of RNA editing, we performed a principal components analysis (PCA) to better characterize the influences of these two factors (Fig. 3). PC1 is correlated with tissue type and PC2 is correlated with acclimation temperature. This analysis indicates that brain showed the highest temperature dependence in editing among the six tissues (Fig. 3, right panel), in addition to being the tissue with the highest amount of editing overall (Fig. 2A, B). Two other tissues, skin and gill, also showed different amounts of editing at different levels of cold challenge (Fig. 3, left panel) as well as the highest number of shared edited sites among the tissues (Fig. 2A).

To further examine inter-tissue differences in response to acclimation temperature, we used PCA to compare gene expression and RNA editing differentiation across tissues from differently acclimated zebrafish. Based on the gene expression data (fragments per kilobase of transcript per million mapped reads, FPKM), principal components analysis of transcriptome data was performed (Additional file 1: Figure S6). The results indicated that PC1 of gene expression is also correlated with tissue type. However, the sample-to-sample distance in the PC1 of gene expression and the PC1 of RNA editing are different. Furthermore, PC2 of gene expression is not correlated with acclimation temperature. In addition, the PCA results show that the changes in gene expression levels in the brain at different temperatures are not as significant as changes in RNA editing.

### Temperature-dependent differential editing sites

**Fig. 4:**
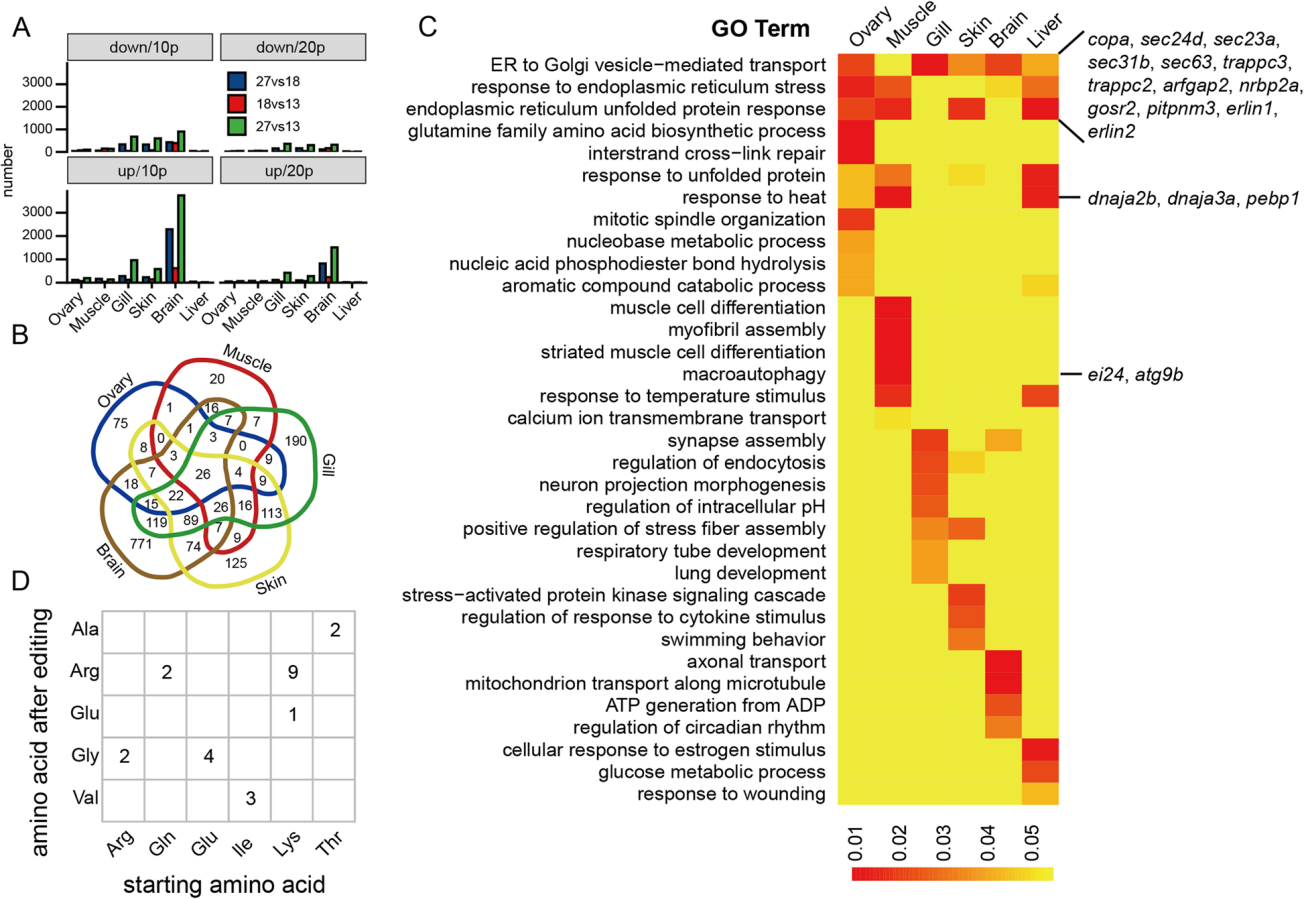
Differential A-to-I RNA editing sites and their related function. **A** Variation in the number of differential RNA editing sites across tissues and temperatures with reduced (down) and increased (up) sites shown separately. 10p and 20p indicate 10 or 20% difference, respectively (T27 compared with T18, T27 compared with T13 and T18 compared with T13). **B** A Venn diagram of differential RNA editing sites involving genes that have 10% differences (10p) in editing. **C** Gene Ontology analysis of differential RNA editing-related genes. **D** An amino acid substitution matrix showing the detected amino acid changes after RNA editing, with the number of each type of substitution shown

The RNA editing sites that differed between any two temperatures were defined as “Temperature-dependent differential editing sites.” We performed a further analysis of temperature-dependent editing to elucidate how the two lower temperatures, 18 °C and 13 °C, influenced the editing process in terms of the number of different editing sites during acclimation to reduced temperatures. The effects of temperature on the number of different RNA editing sites in brain is relatively high compared to other tissues (Fig. 4A). Because expression of genes encoding variants of ADAR could influence responses to temperature, we examined expression levels of four ADAR-encoding genes. In brain, the expression levels of *adar*, *adarb1a*, *adarb1b*, and *adarb2* were slightly but non-significantly (*P* > 0.05) increased under cold stress (Additional file 1: Figure S7). Thus, to a first approximation, temperature effects on ADAR expression do not appear to underlie acclimation-induced effects on amounts of editing.

As might be predicted, the span separating two temperatures positively correlated with the number of differential RNA editing sites in some tissues (Fig. 4A). We identified sites that were presented differentially in the 18 °C and 13 °C acclimated fish relative to the 27 °C group. We termed these sites “temperature-sensitive” A-to-G RNA editing sites. Using this definition, we found that acclimation to 13 °C was accompanied by more RNA editing sites compared to acclimation to 18 °C (Fig. 4A). One of three pairwise comparisons (27 °C vs 18 °C, 27 °C vs 13 °C, or 18 °C vs 13 °C) among the three temperatures revealed differentially edited sites that were identified as “temperature-sensitive”. We next examined the overlap of “temperature-sensitive” RNA editing site-related genes in different tissues (Fig. 4B). A Venn diagram illustrates the amounts of “temperature-sensitive” editing in six tissues.

### Functional roles of proteins encoded by edited mRNAs

Like all ectotherms, fishes are susceptible to thermal stress from both low and high temperatures. Low temperatures that cause cold stress affect diverse biological processes including development, growth, reproduction, locomotion, and others [24, 31]. To see how the edited RNA changes could influence biological function, we utilized Gene Ontology (GO) analysis to gain insights into the functional significance of the RNA editing patterns observed in the six tissues of the specimens acclimated to 18 °C and 13 °C. As shown in Fig. 4C, the GO categories of genes that exhibited significant amounts of RNA editing differed among tissues. For example, in brain high levels of editing occurred in mRNAs encoding proteins related to axon transport, whereas in ovary and muscle editing was high in mRNAs encoding proteins of mitotic spindle organization and muscle cell differentiation, respectively. However, it is notable that genes categorized as belonging to the sets “ER to Golgi vesicle–mediated transport,” “response to endoplasmic reticulum stress,” and/or “endoplasmic reticulum unfolded protein response” all showed editing in all tissues. Another common, albeit not ubiquitous response, was editing of mRNAs encoding proteins associated with processing of mRNA: “response to heat”. Some fractions of the tissue-specific patterns in editing of course are likely to be a consequence of tissue-specific patterns of gene expression, i.e., each tissue would be expected to have a distinct transcriptome. However, the widespread editing among tissues in genes associated with various ER stress responses indicates that RNA editing is likely to play important roles in cold acclimation. These roles could include temperature-adaptive changes in protein sequence and modifications in mRNA stability that improve mRNA function at the new acclimation temperature. While over 97% of editing of mRNAs occurred outside the coding region, we found a total of 23 amino acid substitutions in 13 proteins (Additional file 3: Table S1). We identified substitutions of 6 types of amino acids by 5 another types (Fig. 4D). Among these substitutions, the most frequent editing occurred in the Lys codons, which were transformed to code for Arg (9 cases) and Glu (1 case), respectively. Two types of substitutions (Lys to Arg and Gln to Arg (11 cases)) increased the size of the amino acid, while in another four (total 11 cases), a larger sized amino acid was replaced by a smaller one (Arg by Gly, Glu by Gly, Ile by Val, and Thr by Val), and one case showed almost equal molecular size before and after editing (Lys to Glu). While the cases of size changes appeared almost equal in the two directions, a peculiar phenomenon is the biased gain of Arg (11 cases) and Gly (6 cases). It has been shown that GC-biased codons which included Arg and Gly are evolutionarily favored in cold-water fishes, and Gly is the only favored amino acid substitution in the Antarctic notothenioids compared with warm water fish species [32]. It appears that RNA editing could be a post-transcriptional mechanism to increase the ratio of G-biased codons adaptive to a cold environment. Furthermore, the increased presence of Arg and Gly in the proteins would have the potential to alter thermal sensitivities of the encoded proteins [33].

### Validation of “temperature-sensitive” RNA editing sites detected by in silico analysis

**Fig. 5:**
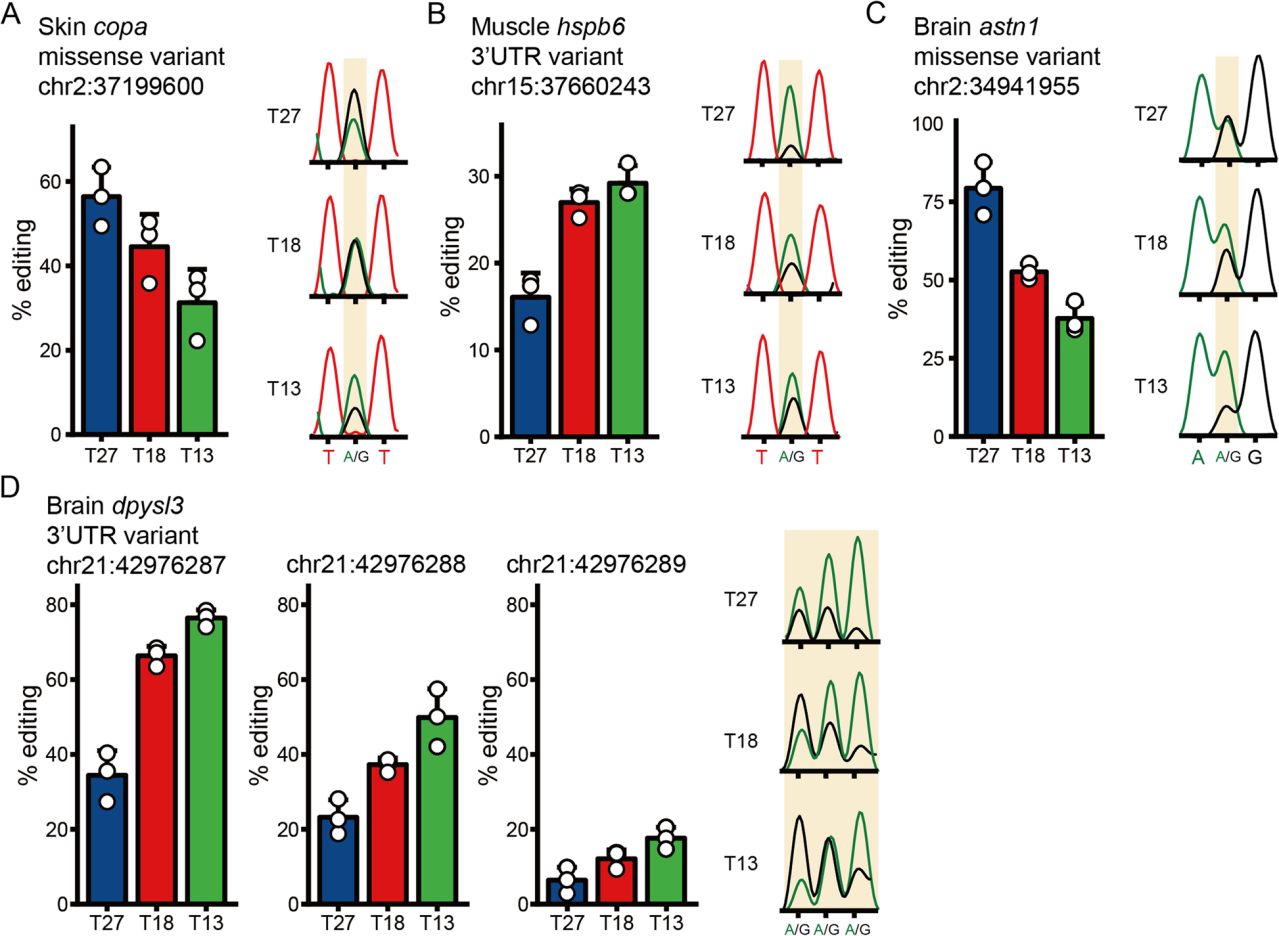
Validation of the dynamic change of RNA editing sites by Sanger sequencing. For each panel, the left bar graph indicates the editing level detected by the in silico method for the three temperatures (Data are shown as the mean ± SD, *n* = 3), while the right graphs indicate the editing levels inferred from Sanger sequencing for each temperature. In the right graphs, the blue line represents guanosine (G) and the black line represents adenosine (A)

To assess the validity of the RNA editing sites, we identified by the in silico methods, we selected 6 sites from the set of differential A-to-I RNA editing sites for validation across tissues and gene feature (3′UTR, CDS) by Sanger sequencing. In the Sanger sequencing chromatograms, RNA editing sites appeared as mixed A and G peaks (Fig. 5A–D). Comparison of the levels of editing sites in RNA-seq and Sanger sequencing shows a high correlation between the two methods, even in adjacent editing sites (Fig. 5D).

### RNA editing increases 3′UTR polymorphism and affects efficiency of translation

**Fig. 6:**
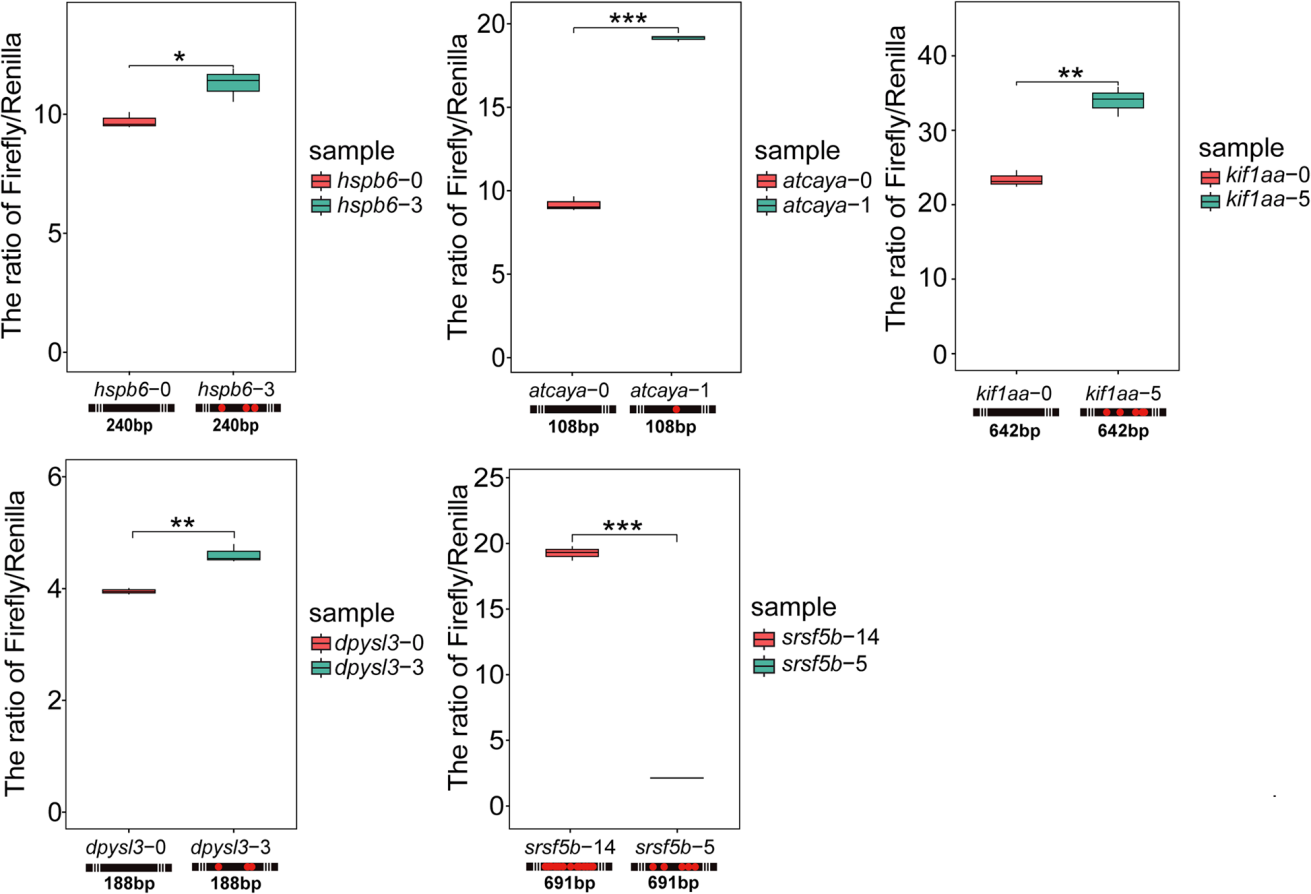
The relationship between the number of edited sites and efficiency of protein synthesis. The ratio of firefly (test) to *Renilla* (background) synthesis (measured as luminescence) detected in the 5 mRNA species indicates efficiency of protein synthesis. Data for warm-preference editing pattern are shown by the left-side bars in each frame; data for cold-preference editing pattern are in the right side of each frame. The number of edited sites in the fragment of the 3′UTR linked to the firefly luciferase reporter gene is shown in the name of the gene after the dash symbol. The bar under each mRNA indicates the length and the locations of edited sites (in red). Significance testing was performed by Student’s *t*-test. *n* = 3, *P < 0.05, **P < 0.01, ***P < 0.001

Because of the high fraction of ADAR editing found in the 3′UTR, a component of mRNA that can be important in governing protein synthesis, we examined the functional significance of editing in the 3′UTR by using the dual luciferase activity assay to measure protein synthetic activity for 5 selected mRNAs exhibiting different temperature-related editing responses in their 3′UTR regions (Fig. 6). The ratio of firefly luciferase activity (measured as luminescence) to *Renilla* luciferase activity indicates the efficiency of protein synthesis, with a high ratio indicating high production of protein. Among the 5 mRNAs selected, four (*atcaya*, *dpysl3*, *hspb6*, *kif1aa*) showed higher RNA editing rates at colder temperature and one (*srsf5b*) showed the opposite trend. Interestingly, in all of the 5 mRNA pairs tested, higher protein translation efficiencies (higher firefly: *Renilla* luminescence ratios) were found in the one that possessed the higher number of edited sites. Furthermore, in HEK-293 T cells (Additional file 1: Figure S8), the effect of editing on efficiency of translation was the same as in zebrafish embryos. The combined results of the dual luciferase assay (Fig. 6) and quantitative real-time PCR (Additional file 1: Figure S9) demonstrate that the dual luciferase assay can directly reflect the efficiency of translation of *atcaya*, *dpysl3*, *hspb6*, and *kif1aa*, because results of quantitative real-time PCR of these four genes pairs showed no significant differences. Although the result of quantitative real-time PCR of *srsf5b* 3′UTR pairs had a significant difference, the ratio of the dual luciferase assay (7.49) far exceeded the ratio of QPCR (1.46).

## Discussion

### Tissue-specific patterns of RNA editing

Our study of temperature effects on editing in six tissues of zebrafish shows that a high level of tissue specificity occurs in total amounts of editing and in the sites that are edited. Both features of editing exhibited strong dependence on acclimation temperature, with reductions in temperature generally but not invariably leading to increased editing of a message, as shown in Fig. 6. Furthermore, temperature effects on editing differed among tissues. The causes and functional consequences of these temperature-dependent tissue-specific changes in RNA editing are largely unknown. In terms of causal relationships leading to tissue-specific editing, some of the observed differences in editing could simply be due to differences in the transcriptomes of the six tissues. In addition, differences might arise from the activities of different isoforms of ADAR that are active in different tissues. Vertebrates typically have three isoforms of ADAR, and these have different tissue distributions and preferences for editing sites [12]. ADAR1 is expressed in all tissues. ADAR2 is expressed most highly in brain but is found in other tissues as well. ADAR3 is expressed exclusively in brain. Because of gene duplication, zebrafish have four ADAR enzymes: ADAR1, ADAR2a, ADAR2b, and ADAR3 [13]. To our knowledge, comparisons of the temperature dependencies of activities or site specificities of the vertebrate ADAR isoforms have not been made. However, in *Drosophila*, which has only a single isoform of ADAR, editing activity at 35 °C was substantially reduced relative to activities at 15 °C [14]. A similar temperature dependence was reported in a later study of *Drosophila* [15]. This reduced activity at high temperature was due to combined effects of a reduced level of expression of the *adar* gene and temperature-dependent auto-editing of the enzyme that led to a reduced level of activity [14]. Temperature effects on ADAR activity could also result from alterations in RNA secondary structure, as discussed below. Further exploration of temperature-ADAR interactions is warranted in order to enhance our understanding of the mechanistic basis of temperature-dependent RNA editing.

The functional consequences of tissue-specific temperature-dependent editing are largely unknown. However, the categories of mRNAs edited in different tissues reflect in some measure effects related to tissue-related functions. Thus, in the highly edited brain, editing of mRNAs associated with synaptic assembly, axonal transport, and regulation of circadian rhythm are consistent with adaptive modification of neural function during acclimation. Studies of temperature acclimation in fish have revealed substantial changes in the biophysical properties of brain synaptic membranes that are correlated strongly with adaptive changes in behaviors like hyperexcitability and maintenance of equilibrium [34]. Studies of invertebrate species also have discovered relatively large amounts of editing in brain, and some of these are recoding events that foster temperature-adaptive changes in protein function. For example, comparisons of polar and tropical octopus species revealed temperature-adaptive recoding of the mRNA of a potassium voltage-gated ion channel that adaptively altered the channel’s function [1, 35]. Increased editing at low temperatures led to a protein with an intrinsically higher rate of channel closing, which would be compensatory for the effects of cold on rate of channel function. It is noteworthy that RNA editing by ADAR most frequently replaces a large side-chain (R group) with a relatively small one [1]. RNA editing thus leads to increased contents of glycine and alanine, for example, which would increase protein flexibility and thereby compensate for effects of reduced temperature on protein conformational stability. RNA editing led to only a relatively small amount of recoding in the cold-acclimated zebrafish (Fig. 4D). In keeping with earlier studies [1], glycine increased during cold acclimation, a change consistent with temperature-compensatory change in protein stability [1, 36]. The adaptive function, if any, of the substitution of arginine for lysine remains to be determined. Studies of temperature-dependent editing in *Drosophila* also have provided evidence for adaptive change in brain function mediated by ADAR-driven editing [4, 15, 17]. The best understood instances of RNA editing in nervous tissue are from studies of mammals, especially humans [1], but temperature effects have not been studied in these homeothermic animals. The many clear examples of how editing-induced changes in amino acid sequence modify the activities of neural proteins like ion channels in homeothermic mammals suggest that brain tissues of ectotherms also may benefit from recoding events that enable these key proteins to function well at different temperatures. In fact, it could be instructive to examine the ectothermic orthologs of mammalian brain proteins that are strongly edited. Such studies might reveal the taxonomic breadth of specific types of editing changes that can affect, for example, channel activity.

In muscle, a considerable amount of editing was in mRNAs encoding proteins involved in muscle cell differentiation, including striated muscle differentiation. Temperature acclimation in fish commonly leads to alterations in capacities for swimming and muscle phenotype, as noted in a study of zebrafish [31]. Thus, some of the editing in classes of RNA like “calcium ion trans-membrane transport” and “myofibril assembly” could reflect temperature-compensatory changes in muscle functional capacities to support swimming and for supporting muscle fiber differentiation, respectively. In gill tissue, editing of mRNAs associated with “regulation of intracellular pH” could reflect adaptive change in properties of systems of acid–base regulation, which might be important in establishing the appropriate temperature-dependent values of extra- and intracellular pH [36]. In mammals, there is evidence for regulation of editing by intracellular acidification [37]. Further investigations of interactions between temperature, RNA editing, and pH regulation are warranted. Likewise, the editing categorized as “response to temperature stimulus” and “response to heat” that occurred in some tissues points to additional lines of future study.

### Similarities in editing activities in different tissues

Whereas there was marked variation among tissues in editing patterns and temperature sensitivity of editing, there were also cases where similarities among tissues were striking. The editing of mRNAs related with “ER to Golgi vesicle–mediated transport” and/or “response to endoplasmic reticulum stress” occurred in all tissues. The results are consistent with previous studies that showed that cold enhanced endoplasmic reticulum stress [38]. Such stress may reflect the extreme sensitivities of membrane biophysical state (static order and propensity to form non-lamellar structures) and, hence, membrane function to changes in temperature [36].

### Temperature effects on RNA structure and amounts of editing

There are a number of reasons to expect that temperature will have a pronounced effect on RNA editing processes. First, the secondary and tertiary structures of RNA are thermally labile, such that even small changes in temperature are likely to perturb these structures [19, 39]. Second, and following from the previous point, because RNA editing by ADAR requires that RNA be double-stranded, temperature changes that either stabilize (lower temperatures) or disrupt (higher temperature) dsRNA could alter the availability of suitable substrates for ADAR activity. To a first approximation then, reduced temperature would favor increased editing due to the availability of more regions of double-stranded structure. We usually found that the amount of editing rose as acclimation temperature was reduced, but as discussed above, there were exceptions to this general pattern.

The most common pattern of temperature dependence of editing was the increase in edited sites that accompanied acclimation to reduced temperature. For example, in brain the number of edited sites rose from 24,678 at 27 °C to 32,471 at 18 °C and to 34,400 at 13 °C. The enhanced level of editing at reduced temperature is consistent with expectations based on the need of ADAR activity for dsRNA. As noted above, the levels of dsRNA would be expected to rise as temperature falls due to more stable base pairing at low temperature. However, this direct effect of temperature on levels of dsRNA, while likely to play some role in determining the temperature dependence of editing, cannot fully account for inter-tissue differences in editing levels or for the variation in editing sites among tissues (Fig. 3).

In the principal components analysis of RNA editing (Fig. 3), the missing values are imputed using missForest [40]. However, it is important to note that the editing levels exhibit a heavy right-skew, which means that imputation of missing values could potentially introduce bias.

### Editing as a means of influencing efficiency of translation and re-organizing the proteome

The discovery that editing affects the efficiency of translation and does so in ways that differ among types of mRNAs (Fig. 6) has major implications for a potential role of editing in re-organizing the proteome during acclimation to temperature. It has been clear for many years that the relative as well as the absolute activities of different biochemical pathways may shift during thermal acclimation in ectotherms [41, 42]. Whereas these shifts may result from temperature-acclimatory alterations in patterns of transcription of new mRNAs [42], our finding that editing of 3′UTRs leads to significant changes in the efficiency of translation and that these effects differ among classes of mRNA points to another mechanism for acclimation of the proteome and metabolic re-organization. Importantly, the finding that one of the five mRNAs examined (*srsf5b*) exhibited greater editing at high (18 °C) than low (13 °C) temperatures illustrates the potential versatility of ADAR editing in orchestrating re-organization of the proteome, whether acclimation temperature is increased or decreased. The role of editing on the 5′UTR sequence could also be important in governing translation, even though much less editing occurred in this region relative to the 3′UTR. Because the 5′ region is key for initiation of translation, even small changes in base composition that lead to alterations in conformational stability could be instrumental in adjusting rates of initiation [43].

The mechanisms by which editing of the 3′UTR altered the efficiency of translation of these five mRNAs were not examined in our study, but there is evidence to support the conjecture that editing of UTRs that alters their secondary structures might account for these effects. Studies in *Drosophila* have shown that high fractions of edited sites (at 18 °C) are in regions of secondary structure [15]. Moreover, editing at a single base has been shown to be sufficient to alter RNA secondary structure [15]. When such editing occurs in UTRs, it could be important in governing different events in the process of translation. Thus, the secondary structures of 5′ and 3′UTRs are known to play important roles in modulating key steps of translation, including rates of initiation and elongation. For example, reduced secondary structure in the 5′UTR region enhances initiation, whereas increased secondary structure in the CDS and 3′UTR favors high protein expression [44]. Our finding that increased editing of the 3′UTRs in 5 different mRNAs led to higher efficiency of translation might be a reflection of increased secondary structure in these regions due to editing, increases that occur in response to either a rise or fall in acclimation temperature, depending on the mRNA in question.

The time courses of editing-mediated changes in the transcriptome and proteome merit further consideration. Rapid changes in the transcriptome in response to an acute change in temperature may involve differential effects on the 3′UTRs of different classes of mRNAs. For example, a rapid and adaptive re-organization of the transcriptome in response to acute change in temperature was demonstrated by Su et al. [45]. Their work (with rice) showed that disruption of 3′UTR structure by acute increases in temperature fostered degradation of “housekeeping” mRNAs by making the RNAs susceptible to RNA degradation. This effect was not found in mRNAs that encode stress-related proteins [20]. The roles of such temperature effects on 3′UTR regions during longer-term acclimatory processes remain to be determined. However, these findings suggest that RNA editing that occurs in 3′UTR sequences and influence mRNA stability and turnover could be important in adjusting the composition of the transcriptome and, therefore, the proteome during temperature acclimation. Proteome re-organization mediated through editing-governed translational efficiency would likely be more quickly achieved than effects requiring transcription because editing effects could arise from rapid alterations of translational activities involving pre-existing mRNAs. Thus, editing of existing transcripts might contribute importantly to at least the initial shifts in metabolic organization that occur during acclimation. Although the effects of acute temperature changes on 3′UTR secondary structures are well established, the roles of ADAR editing in adjusting the stabilities of 3′UTRs over longer acclimation periods such as the 30-day acclimation period of the present study remain to be demonstrated.

Although the work performed with the dual luciferase system involved only five types of mRNAs, the consistency in the findings—translation was stronger in the edited mRNAs in all cases—points to the possibility of a general role of mRNA editing in altering translational patterns in the face of changes in temperature. These ADAR-mediated effects could be important in responses to both decreases and increases in temperature, as shown by the data in Fig. 6. This discovery raises interesting questions about differences among RNAs in temperature sensitivities in their ADAR sites that determine whether editing is favored by increase or decrease of temperature. Thus, do ADAR sites evolve to have specific responses to increases or decreases in temperature that lead to adaptive changes in gene (protein) expression, as mediated through translational efficiency? Do the effects of editing modify the secondary or tertiary structures of the mRNAs in manners that influence initiation of translation, rates of translation, and mRNA half-life? Answers to these questions would shed important light on the mechanisms by which ectotherms re-organize their metabolic pathways during thermal acclimation.

A final point to consider in the context of how mRNA editing might lead to metabolic re-organization during temperature acclimation is the finding that acclimation temperature has an effect on mRNA splicing. Healy and Schulte [46] discovered that cold acclimation affected splicing patterns in hundreds of genes in four species of fishes. A number of the genes for which temperature-dependent splicing was observed were genes that exhibited upregulation in the cold. It is not known if RNA editing was the mechanism involved in governing alternative splicing, but this possibility merits investigation [9]. Thus, it is possible that mRNA editing could alter the proteome by changing splicing patterns as well as by modulating translational efficiencies of mRNAs.

### CDS editing: roles of synonymous base changes

Whereas the importance of recoding editing in modifying the thermal responses of proteins has been demonstrated [1], the roles of synonymous changes in the CDS remain to be analyzed in the context of thermal acclimation. Because changes between synonymous codons can affect rates of translation [47] and the final folded state of a protein [48], even the small fraction of synonymous changes noted in the CDS could have physiological significance through affecting the amounts of protein synthesized and the final conformational and functional states of the proteins.

### Conservation of corresponding states of stability in RNA secondary and tertiary structures—a major role for RNA editing in ectotherms?

Lastly, we wish to consider issues that reflect major differences between editing in homeothermic endotherms like mammals and ectothermic species like fishes. We conjecture that ADAR editing may play quite different roles in these two broad groups of animals. To approach these differences, we first consider another perspective on the significance of temperature-dependent stability of the base pairing that establishes RNA secondary and tertiary structures. This analysis is based on considerations of the need to conserve the abilities of RNAs to undergo reversible conformational changes that are essential for function at different body temperatures. Reversible formation of secondary structural elements is key to several aspects of mRNA function, so the ability to retain a capacity for undergoing these reversible changes in conformation at different temperatures seems critical. This type of conservation of conformational stability is found in orthologous proteins of species adapted to different temperatures [19, 49, 50] and is termed the retention of “corresponding states.” The recent discovery of evolved temperature-adaptive differences in stability of mRNA secondary structure suggests that mRNA structure, like protein structure, evolves to maintain the correct balance between conformational stability and flexibility, i.e., corresponding states of conformational stability, at normal cell temperatures [21].

We conjecture that a similar temperature-adaptive pattern in RNA stability could arise from RNA editing and could potentially conserve the right balance between stability and flexibility in all components of mRNA (CDS, 3′UTR, introns, etc.). Increased strength of base pairing as temperature is reduced could lead to perturbation of RNA function if, for example, secondary structures become too rigid to allow needed changes in mRNA conformation during translation, splicing reactions, or RNA turnover. Editing might lead to reduced amounts of base pairing at low temperature, an effect that could compensate for the strengthening of base pairing due to reduced thermal energy. This type of adaptive change in RNA structural stability across different genomic regions during acclimation may be the most general role of temperature-dependent RNA editing in ectotherms. Conversely, the fact that the preponderance of RNA editing in mammals is focused on repetitive sequences, e.g., Alu elements, and is less common in other genomic regions, could be a sign of a lack of need for genome-wide temperature-compensating changes in RNA stability in homeotherms. In this context, it merits mentioning recent work on hibernating (heterothermic) ground squirrels in which RNA editing increased during periods of low body temperature [7]. Few changes in CDS were observed; most editing was in interspersed repeats (SINE elements) that engage in dsRNA formation and can trigger the innate immune response [51]. Enhanced RNA editing during hibernation may be a mechanism for minimizing inflammation during periods at low, near-zero body temperatures [7], rather than a mechanism for ensuring corresponding states of RNA structural stability to allow maintenance of all categories of RNA function.

## Conclusions

In summary, we conjecture that A-to-I editing plays a number of roles in acclimation to temperature in ectotherms and endotherms, and that these roles may differ in important respects between the two groups. Temperature-compensatory adjustment in RNA stability could be important in ectotherms, to ensure that reversible changes in RNA secondary and tertiary structure can occur. These compensatory changes in stability may be important components of a broader function for ADAR-catalyzed editing in orchestrating adaptive re-organization of metabolic pathways during temperature acclimation.

## Methods

### Zebrafish culture and RNA/DNA extraction

To identify the temperature-dependent editing events, nine adult wild type zebrafish (*Danio rerio* AB strain, 6 months) held at 27 °C were divided randomly into three groups of three fish and transferred into three equivalent recirculating aquaculture systems set at 27 °C, 18 °C, and 13 °C. Acclimation lasted 30 days. Only females were used to control for possible sex-specific differences. The epaxial skeletal muscles, gills, brains, skins, livers, and ovaries of all nine specimens were dissected from the bodies with scissors and tweezers for RNA extraction. The residual tissue was used for DNA extraction. Total RNA was extracted using Trizol reagent (Sigma). Using the method of protease K-phenol chloroform, the DNA was extracted from the nine specimens.

### Library construction and sequencing

All strand-specific RNA-seq libraries were constructed using VAHTS Stranded mRNA-seq library Prep kit for Illumina v2 (Vazyme Biotech Co., Ltd, NR612-02) and subjected to 150 bp paired-end sequencing on a Novaseq 6000 Illumina platform. To decrease the cost of constructing libraries and sequencing, we pooled equivalent amounts of the genomic DNA extracted from the nine specimens. The DNA-seq library was constructed using an Annoroad Universal DNA library Prep Kit V2.0 (ANNOROAD, AN200101) and was subjected to 150 bp paired-end sequencing on a Novaseq 6000 Illumina platform.

### Detection of editing and hyper-editing in RNA-seq data and comparing RNA-seq and DNA-seq data

We then performed RNA editing analysis using previously published methods (RES-scanner) [25] for normal RNA editing sites and an advanced version (RES-scanner2) for hyper-editing sites [26]. In brief, genome re-sequence reads and transcriptome sequences were aligned using BWA software [52] to the zebrafish reference genome (GRCz10) or transcripts from the Ensembl Genome Browser, only maintaining unique alignment. Other principal rules used to detect the sites of RNA editing are as follows: (1) trimming the first and last six bases of each aligned read for further analysis. (2) Removing all known SNPs extracted from DNA-seq (supported by a sufficient number of reads). (3) Reliable RNA editing sites were identified when there were enough sequencing reads at the sites (Phred quality score ≥ 30). (4) Sites that had two or more variants were excluded, and (5) using BLAT to realign and check all the reads that support RNA editing. In coding regions, the editing type of each mismatch was determined on the basis of the Ensembl annotation (release-85). The functional annotation of the RNA editing sites was inferred with the software snpEff [53] version 4.3t. The Venn diagram was created by the Vennerable package in R. The seqlogo diagram was drawn using the ggseqlogo package [54]. For the analysis of the editing level, we required a minimum coverage of 10 reads.

### Principal components analysis (PCA)

In total, editing levels measured from 54 samples spanning 6 tissues and 3 acclimation temperatures were used for PCA. We averaged the editing levels for samples of the same tissue type from the same temperature. We imputed missing values for the sites using the missForest [40] packages and used the prcomp function in R to perform the PCA.

### Differential RNA editing sites calculation

For multiple samples per group, we calculated the differential RNA editing sites using the logistic regression method integrated into methylKit software [55]. In brief, statistical significance of alteration between temperatures for each editing site also was done using the logistical regression procedure. The differential RNA editing sites were defined by 5% FDR correction and 10 or 20% difference value. The enrichments of GO and KEGG were performed by the Clustalprofiler package [56].

### Microinjection

Male and female fish were placed at an even ratio in breeding traps, where they were held overnight, and eggs were collected 0.5 h after the onset of light and were cultured in water containing 0.5 mg/L methylene blue. The injection mixture contained a final concentration of 25 ng/μL of EGFP-Tol2 plasmid DNA and 25 ng/μL of plasmid DNA. Each egg was injected with 1nL of the mixture. At least 200 embryos were injected for each plasmid. After 24 h development of embryos, we selected eggs with green fluorescence using a fluorescence microscope (Zeiss). We homogenized 90 eggs with each plasmid, adding 150μL DPBS with 1% PMSF for dual luciferase reporter assay.

### Quantitative real-time PCR

Each sample included about 15 injected eggs for total RNA extraction. The total RNA extraction method was the same as above. Reverse transcription used the PrimeScript™ RT reagent Kit with gDNA Eraser (Perfect Real Time) from Takara. Primers were designed by Primer Premier 5. RT-PCR used ChamQ Universal SYBR qPCR Master Mix from Vazyme and was conducted in a BIO-RAD CFX96. The expression level was normalized to hRluc-neo fusion using the 2 − ΔΔCt method. luc2 reporter gene (firefly): forward, 5′-CACCGTCGTATTCGTGAGCAA-3′; reverse: 5′-GGGTGGCAAATGGGAAGTCA-3′. hRluc-neo fusion protein coding region (*Renilla*): forward, 5′-TCTGATGCCGCCGTGTTCC-3′; reverse, 5′-CCCAATAGCAGCCAGTCCCT-3′.

### Recombinant plasmids construction

The *hspb6*, *kif1aa*, *atcaya*, *dpysl3*, and *srsf5b* 3′UTR sequences were amplified by polymerase chain reaction and sequenced. Two fragments with different numbers of edited sites were chosen for cloning and used for dual fluorescence luciferase activity assay. The homologous arms from the pmirGLO vector multiple cloning site (MCS) were added to the PCR product. The PCR products were inserted into the MCS located downstream of the luc2 reporter gene in the dual luciferase reporter vector pmirGLO Vector containing the Green and *Renilla* florescence reporting system (Promega) by In-Fusion® HD Cloning Kit (Takara Bio).

### Cell culture and transfection

The HEK-293 T cells were plated in 24-well plates with a density of 10^6^ cells/well until reaching 70–90% confluence. After that, 500 ng of constructed plasmid per well were transfected into cells using a Lipofectamine6000 transfection kit (Invitrogen). We replaced the previous medium in each well with new DMEM after 6 h. The values of dual luciferase were measured after 24 h post-transfection.

### mRNA translational efficiency using dual florescence luciferase activity assay

The dual luciferase reporter assay used the Dual-Glo® Luciferase Assay System (Promega) and was measured with a BioTek Synergy2 spectrofluorophotometer. To each well of the 96-well flat-bottom plate (Beyotime) was added 75μL egg homogenate and an equal volume of Dual-Glo® Reagent. At a time of at least 10 min after mixing of the egg homogenate and reagent, the firefly luminescence was quantified in a luminometer. Then, a volume of Dual-Glo® Stop & Glo® Reagent equal to the original culture medium volume was added to each well and mixed. After at least 10 min post-mixing, the *Renilla* luminescence was quantified in a luminometer.

### Supplementary Information

**Additional file 1:** **Figure S1. **Relative abundance of each editing type in hyper-editing mode and normal mode.** Figure S2. **The relative abundance of editing in TE and non-TE regions.** Figure S3. **The percentage of editing in various genomic regions. Syn indicates synonymous CDS sites.** Figure S4. **Characteristics of the editing level of each tissue based on the total editing sites combined from all tissues (a total of 298,698 sites).**Figure S5. **Global repeats editing index.** Figure S6. **Principal component analysis (PCA) of FPKM profiles across tissues from zebrafish acclimated to different temperatures.** Figure S7. **Expression level of *adar*, *adarb1a*, *adarb1b* and *adarb2* genes across tissues and temperatures, as measured by FPKM.** Figure S8. **The relationship between the number of edited sites and efficiency of protein synthesis in 293T cells.** Figure S9. **Quantitative real-time PCR for 24h zebrafish embryos which were injected with the structured plasmids.

**Additional file 2:** The editing levels of all the A-to-I sites in each of the tissues at the various temperatures.

**Additional file 3:** **Table S1. **The types of amino acid substitution induced by RNA editing.

**Additional file 4:** The individual data values for Figure. 2B, D-E,5, 6, S4B, C, S5, S7, S8, S9.

## Data Availability

All data generated or analyzed during this study are included in this published article, its supplementary information files and publicly available repositories. The sequencing data have been deposited in SRA at NCBI under the accession number PRJNA846110 [57]. Original data of replicated experiments used for quantification can be found in Additional file 4.

## Abbreviations

ADAR: Adenosine deaminase acting on RNA
CDS: Coding sequence
3′UTRs: 3′ Untranslated regions
dsRNA: Double-stranded RNA
TEs: Transposable elements
lincRNAs: Long intergenic non-coding RNAs
FPKM: Fragments per kilobase of transcript per million mapped reads
GO: Gene Ontology
PCA: Principal component analysis
MCS: Multiple cloning site
