# Supplementary material for: Tissue-specific temperature dependence of RNA editing levels in zebrafish
Source: BMC Biol. 2023 Nov 20;21:262. doi: 10.1186/s12915-023-01738-4 (PMC10659053; doi:10.1186/s12915-023-01738-4)
Supplement: Supplementary file 1 — Additional file 1: Figure S1. Relative abundance of each editing type in hyper-editing mode and normal mode. Figure S2. The relative abundance of editing in TE and non-TE regions. Figure S3. The percentage of editing in various genomic regions. Syn indicates synonymous CDS sites. Figure S4. Characteristics of the editing level of each tissue based on the total editing sites combined from all tissues (a total of 298,698 sites).Figure S5. Global repeats editing index. Figure S6. Principal component analysis (PCA) of FPKM profiles across tissues from zebrafish acclimated to different temperatures. Figure S7. Expression level of adar, adarb1a, adarb1b and adarb2 genes across tissues and temperatures, as measured by FPKM. Figure S8. The relationship between the number of edited sites and efficiency of protein synthesis in 293T cells. Figure S9. Quantitative real-time PCR for 24h zebrafish embryos which were injected with the structured plasmids. [file 12915_2023_1738_MOESM1_ESM.docx]

***Additional files 1***

**Tissue-specific temperature-dependence of RNA editing levels in zebrafish**


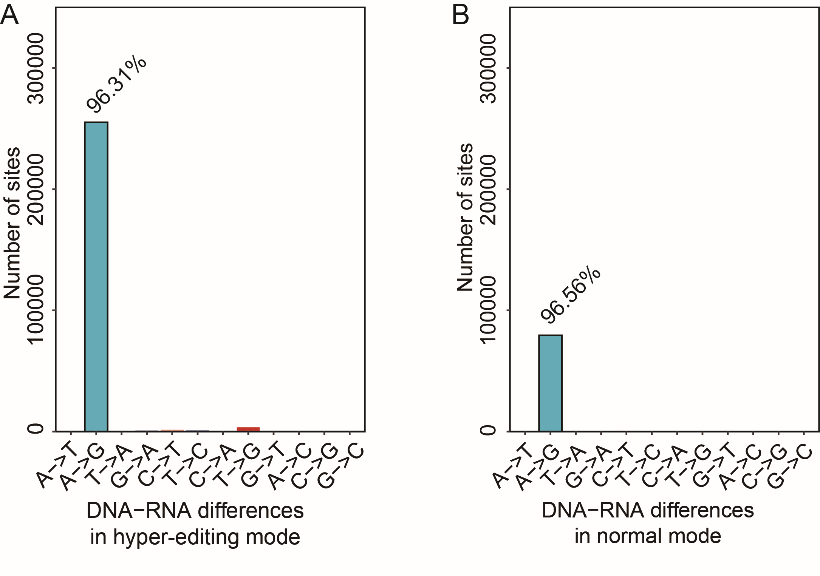


**Figure S1.** Relative abundance of each editing type in hyper-editing mode and normal mode. (A) hyper-editing mode. (B) normal mode.


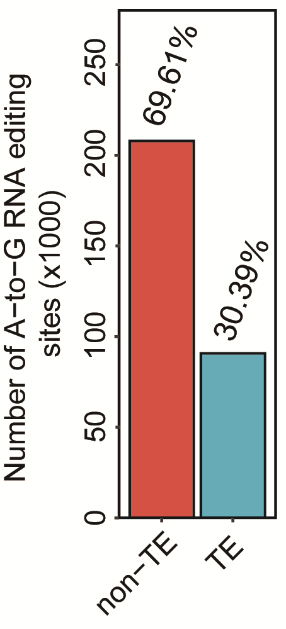


**Figure S2.** The relative abundance of editing in TE and non-TE regions.


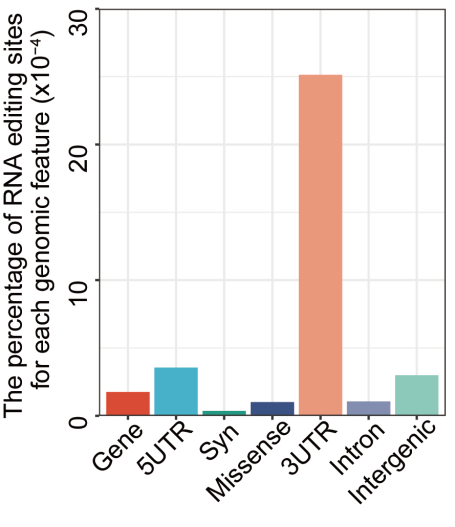


**Figure S3.** The percentage of editing in various genomic regions. Syn indicates synonymous CDS sites.

**
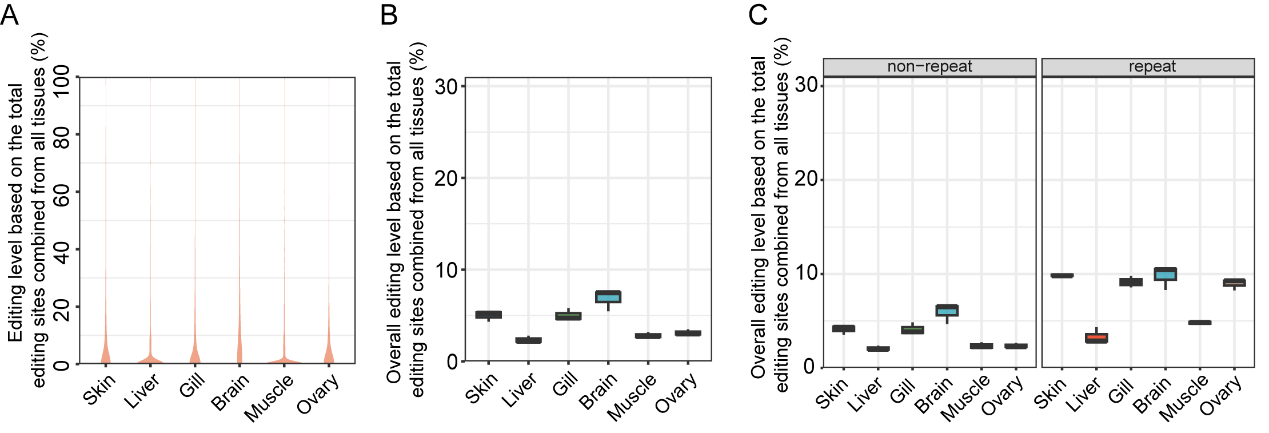
**

**Figure S4.** Characteristics of the editing level of each tissue based on the total editing sites combined from all tissues (a total of 298,698 sites). (A) Editing level of each tissue based on the total editing sites combined from all tissues (%). (B, C) Overall editing level of each tissue based on the total editing sites combined from all tissues (%), and non-repeat DNA regions or repeat DNA regions. For the analysis of the editing level, we required a minimum coverage of 10 reads, n=3.


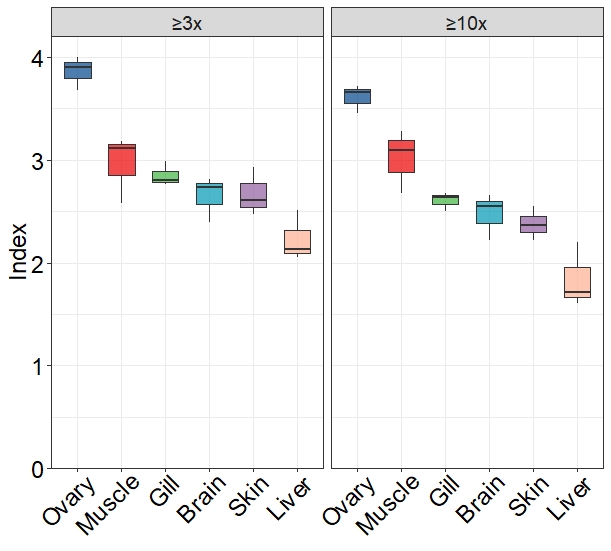


**Figure S5.** Global repeats editing index. The weighted editing level over all adenosines in the edited repeats, as measured in six different tissues. (≥3x means at least 3 RNA seq reads, ≥10x means at least 10 RNA seq reads), n=3.


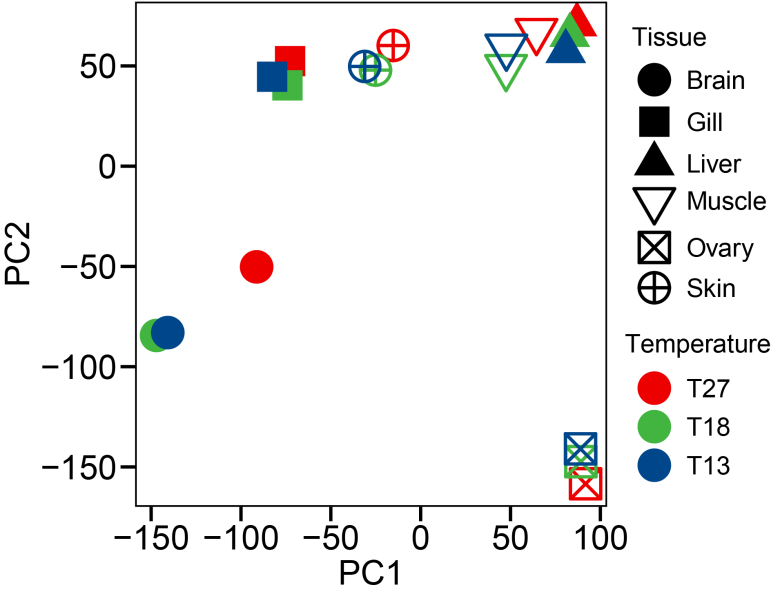


**Figure S6.** Principal component analysis (PCA) of FPKM profiles across tissues from zebrafish acclimated to different temperatures.


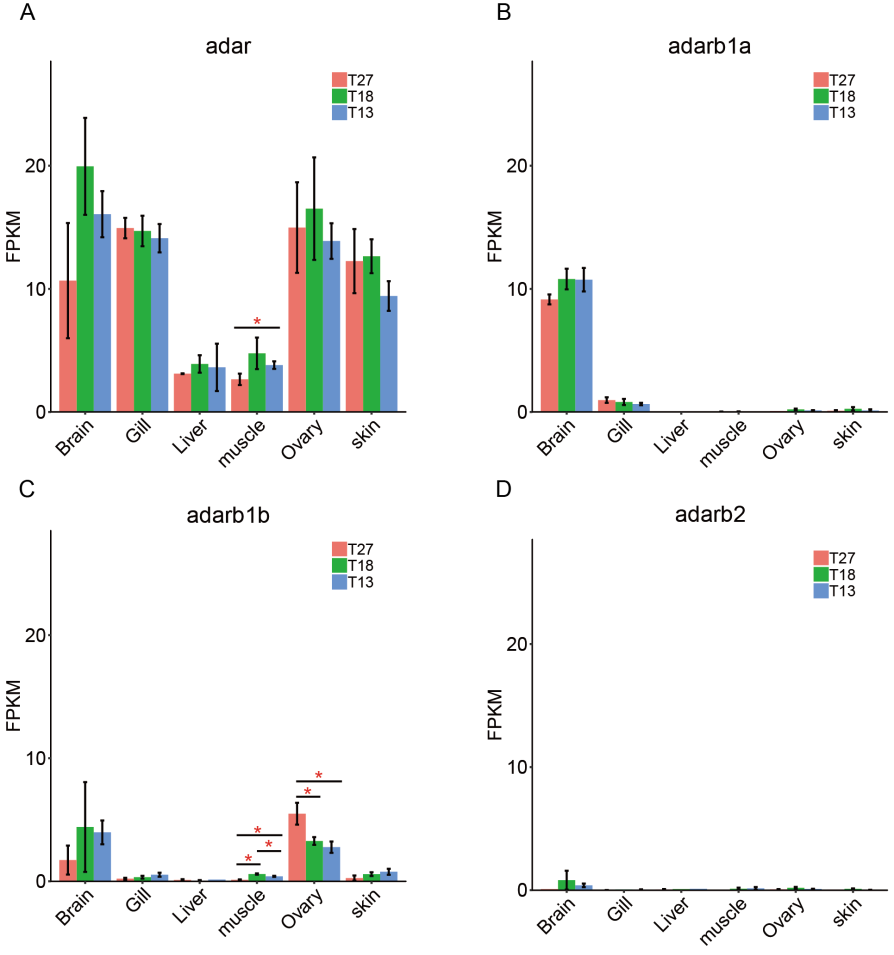


**Figure S7.** Expression level of *adar, adarb1a, adarb1b* and *adarb2* genes across tissues and temperatures, as measured by FPKM. Data are shown as the mean±SE, n=3, *P<0.05.


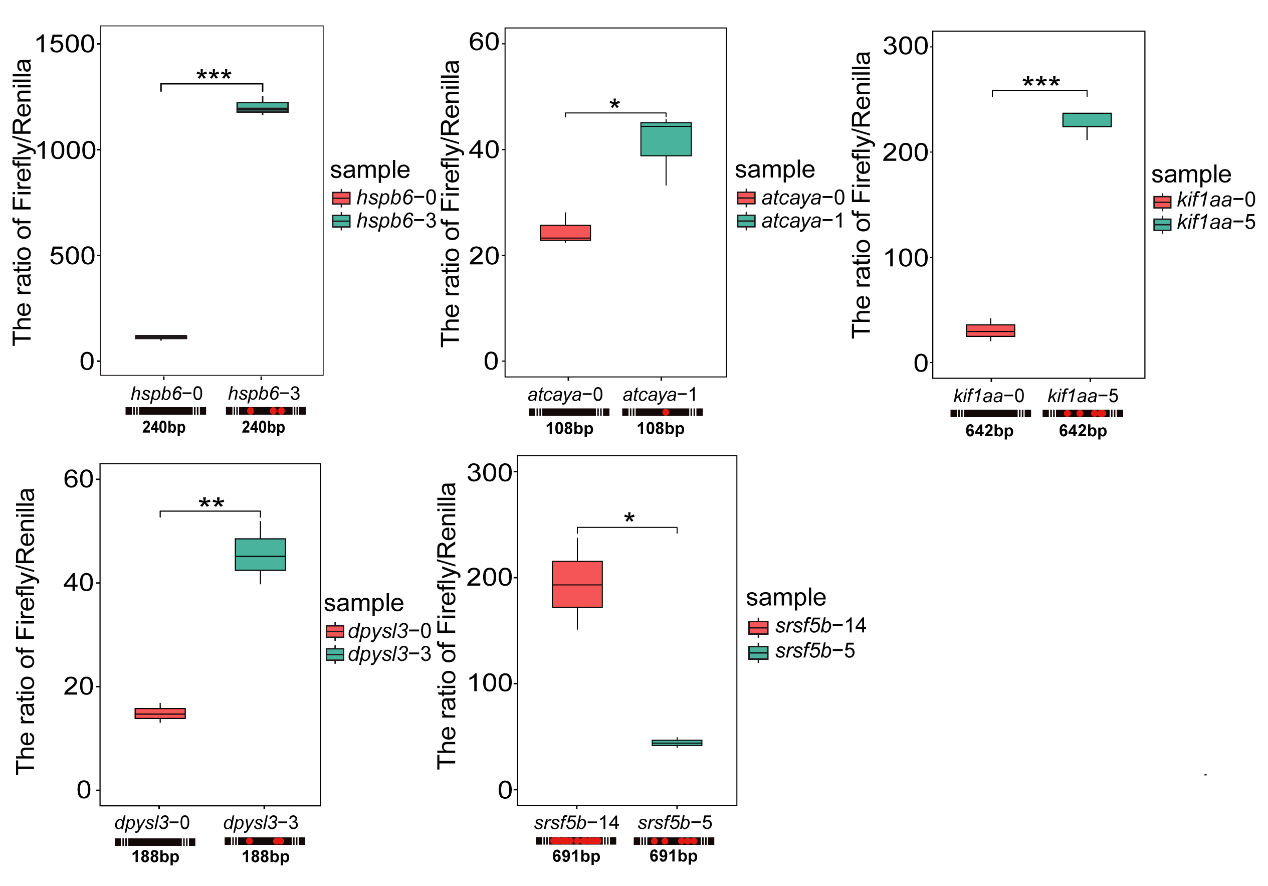


**Figure S8.** The relationship between the number of edited sites and efficiency of protein synthesis in 293T cells. n=3, *P<0.05, **P < 0.01, ***P < 0.001.


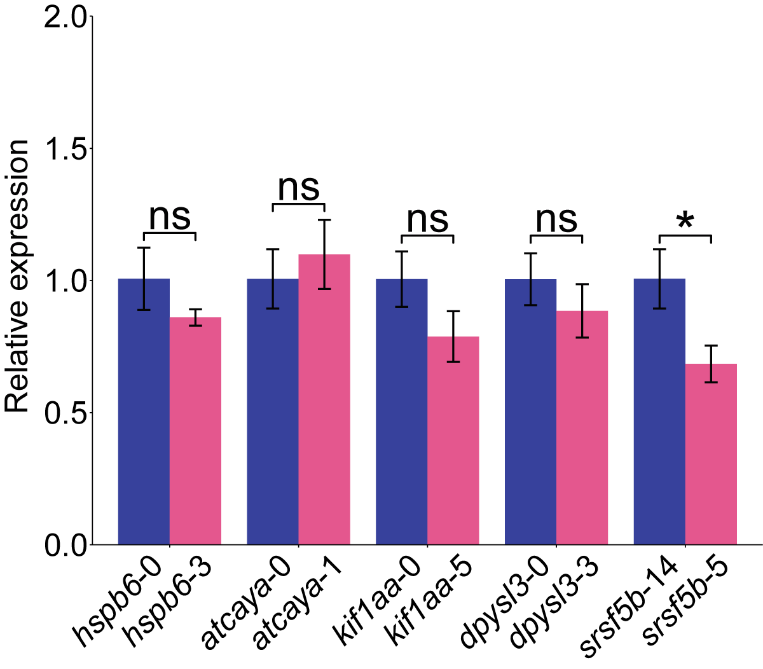


**Figure S9.** Quantitative real-time PCR for 24h zebrafish embryos which were injected with the structured plasmids. Data are shown as the mean±SD, n=3, ns, not significant, *P < 0.05.
